# Histone H3 core domain in chromatin with different DNA linker lengths studied by ^1^H-Detected solid-state NMR spectroscopy

**DOI:** 10.3389/fmolb.2022.1106588

**Published:** 2023-01-04

**Authors:** Sean T. Smrt, Nicole Gonzalez Salguero, Justin K. Thomas, Mohamad Zandian, Michael G. Poirier, Christopher P. Jaroniec

**Affiliations:** ^1^ Department of Chemistry and Biochemistry, The Ohio State University, Columbus, OH, United States; ^2^ Department of Physics, The Ohio State University, Columbus, OH, United States

**Keywords:** chromatin, nucleosome array, histone, linker DNA, magic angle spinning (MAS) solid-state NMR

## Abstract

Chromatin, a dynamic protein-DNA complex that regulates eukaryotic genome accessibility and essential functions, is composed of nucleosomes connected by linker DNA with each nucleosome consisting of DNA wrapped around an octamer of histones H2A, H2B, H3 and H4. Magic angle spinning solid-state nuclear magnetic resonance (NMR) spectroscopy can yield unique insights into histone structure and dynamics in condensed nucleosomes and nucleosome arrays representative of chromatin at physiological concentrations. Recently we used J-coupling-based solid-state NMR methods to investigate with residue-specific resolution the conformational dynamics of histone H3 N-terminal tails in 16-mer nucleosome arrays containing 15, 30 or 60 bp DNA linkers. Here, we probe the H3 core domain in the 16-mer arrays as a function of DNA linker length *via* dipolar coupling-based ^1^H-detected solid-state NMR techniques. Specifically, we established nearly complete assignments of backbone chemical shifts for H3 core residues in arrays with 15–60 bp DNA linkers reconstituted with ^2^H,^13^C,^15^N-labeled H3. Overall, these chemical shifts were similar irrespective of the DNA linker length indicating no major changes in H3 core conformation. Notably, however, multiple residues at the H3-nucleosomal DNA interface in arrays with 15 bp DNA linkers exhibited relatively pronounced differences in chemical shifts and line broadening compared to arrays with 30 and 60 bp linkers. These findings are consistent with increased heterogeneity in nucleosome packing and structural strain within arrays containing short DNA linkers that likely leads to side-chains of these interfacial residues experiencing alternate conformations or shifts in their rotamer populations relative to arrays with the longer DNA linkers.

## Introduction

Eukaryotic DNA is organized into chromatin, a dynamic supramolecular complex with histone proteins that regulates genome accessibility and essential genome functions including transcription and repair (Li et al., 2007; Sinha and Peterson, 2009; Woodcock and Ghosh, 2010; Li and Reinberg, 2011). The basic unit of chromatin is the nucleosome, which consists of ∼147 base pairs (bp) of DNA wrapped approximately two times around a histone octamer complex composed of two copies each of histones H2A, H2B, H3 and H4 (Luger et al., 1997; Zhou et al., 2019) with all histones containing structured, largely α-helical domains making up the nucleosome core and positively charged ∼15–35 amino acid (aa) residue N-terminal tails that extend out of the nucleosome (Zhou et al., 2019). Adjacent nucleosomes in chromatin are connected by linker DNA segments, the length of which varies between ca. 10 and 90 bp, corresponding to nucleosome repeat lengths of ∼157–237 bp (Lantermann et al., 2010; Hughes and Rando, 2014), for different organisms, cell types and cell cycle phases. While the length of these DNA linkers is not necessarily completely uniform (Ricci et al., 2015) it generally does not vary extensively within a given cell type (Grigoryev, 2018), with transcriptionally active eukaryotic cells having relatively short DNA linker lengths (∼40 bp or less) and longer linkers (∼50 bp or more) typically found in mature transcriptionally inactive cells (Perišić et al., 2010). The exact linker DNA length also profoundly impacts the relative orientation of adjacent nucleosomes, chromatin structure and compaction (Widom, 1992; Wang et al., 2008; Correll et al., 2012; Collepardo-Guevara and Schlick, 2014; Grigoryev, 2018), chromatin transcriptional activity (Norouzi et al., 2015), as well as chromatin unfolding (Kruithof et al., 2009; Collepardo-Guevara and Schlick, 2013) and phase separation properties (Gibson et al., 2019; Singh and Mueller-Planitz, 2021). In addition to linker DNA, chromatin structure, compaction and transcriptional activity are also regulated by other factors, including covalent histone post-translational modifications (PTMs) (Strahl and Allis, 2000; Cosgrove et al., 2004; Tropberger and Schneider, 2010; Zentner and Henikoff, 2013; Bowman and Poirier, 2015; Kebede et al., 2015), which modulate the conformational dynamics and interactions of histone tail and core domains and nucleosome plasticity.

While significant progress has been made in atomic-resolution structure determination for nucleosomes, nucleosome arrays and their complexes with chromatin binding proteins by X-ray crystallography and, more recently, cryo-electron microscopy (Zhou et al., 2019; Bai and Zhou, 2021; Takizawa and Kurumizaka, 2022), as well as characterization of conformational dynamics of histone tail and core domains in mononucleosomes by solution-state nuclear magnetic resonance (NMR) spectroscopy (Musselman and Kutateladze, 2022), studies of histone protein structure, conformational dynamics and interactions in condensed nucleosomes and nucleosome arrays representative of chromatin at physiological concentrations come with considerable challenges that can uniquely be addressed by using magic angle spinning solid-state NMR techniques (van Emmerik and van Ingen, 2019; Ackermann and Debelouchina, 2021; Shi et al., 2022). Indeed, these solid-state NMR studies have revealed that histone N-terminal tails retain considerable conformational flexibility even in the highly condensed chromatin environment (Gao et al., 2013; Shi et al., 2018; Xiang et al., 2018; Shi et al., 2020a; Zandian et al., 2021), which likely plays a significant role in recruitment of chromatin regulatory proteins, and enabled histone core dynamics (Shi et al., 2020b), base pairing in nucleosomal DNA (Conroy et al., 2022), histone protein interactions with nucleosomal DNA (Elathram et al., 2022) and with nucleosome binding peptides and proteins (Xiang et al., 2018; le Paige et al., 2021), as well as the impact of PTMs on histone tail conformation and flexibility (Shoaib et al., 2021) to be probed with atomic-resolution detail.

To evaluate the influence of linker DNA length on the residue-specific conformational dynamics and interactions of histone H3 tails in chromatin, we recently performed quantitative ^15^N spin relaxation measurements in large nucleosome arrays consisting of 16 tandem repeats of the Widom 601 nucleosome positioning sequence (Lowary and Widom, 1998) with 15, 30 or 60 bp DNA linkers using J-coupling based MAS solid-state NMR techniques (Zandian et al., 2021). These studies revealed non-uniform local dynamics along the H3 tails, which became increasingly restricted when moving from isolated soluble nucleosomes to the chromatin environment, indicative of transient electrostatic interactions of positively-charged H3 tail residues with linker DNA, and, remarkably, the H3 tail conformational dynamics were found to be relatively insensitive to the precise length of the DNA linkers within the 15–60 bp range investigated. Here, we investigate the 16-mer nucleosome arrays with different DNA linker lengths by using ^1^H-detected dipolar coupling-based MAS solid-state NMR methods to assess the impact of linker length in chromatin on the conformation and flexibility of the histone H3 core domain.

## Results and discussion

### Fingerprint solid-state NMR spectra for histone H3 core domain in nucleosome arrays

**FIGURE 1:**
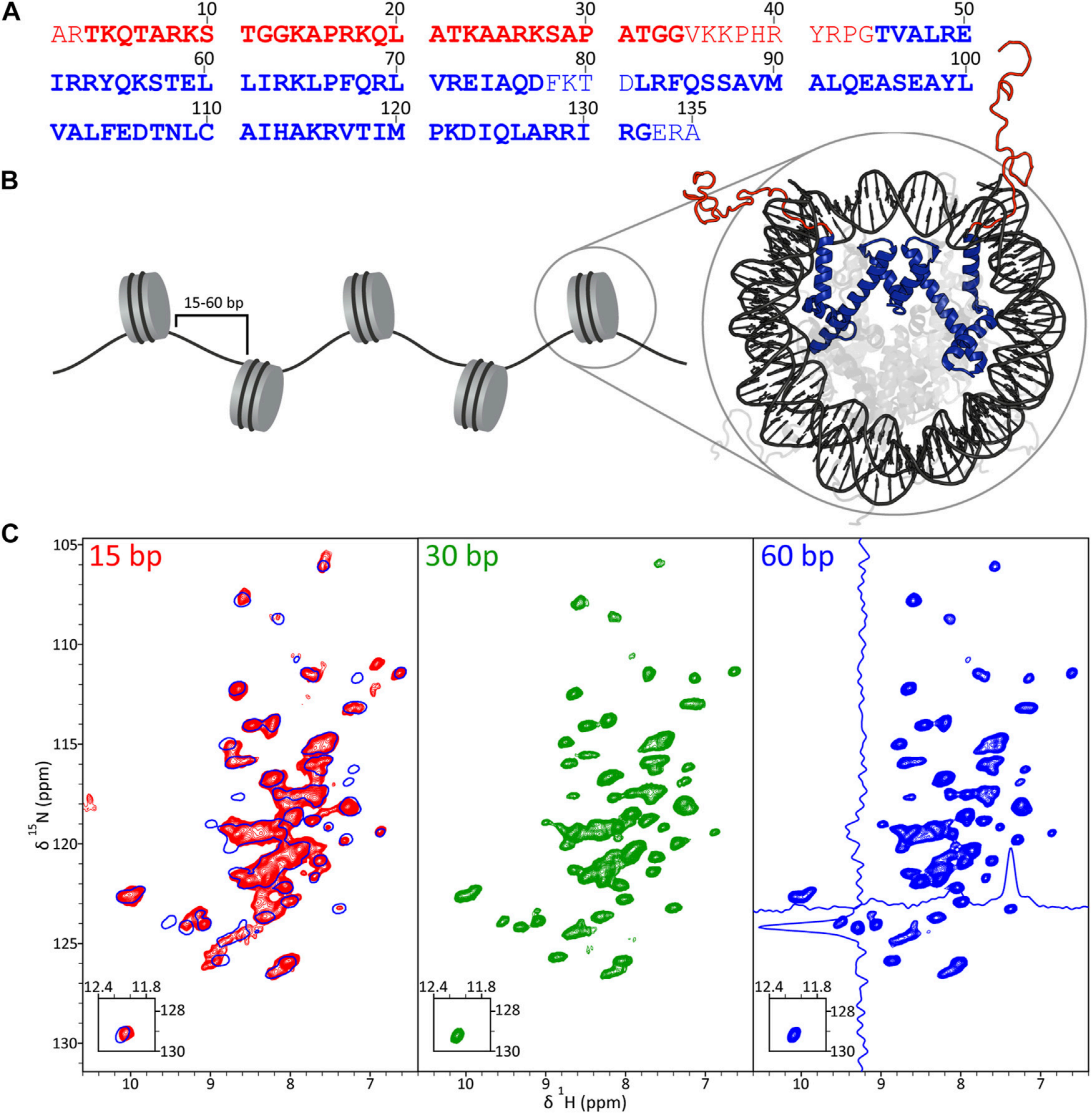
**(A)** Primary sequence of *Xenopus laevis* histone H3, with disordered tail and structured core domain residues indicated in red and blue font, respectively, based on the nucleosome crystal structure (PDB 1KX5). Also highlighted are residues detectable in solid-state NMR experiments employing J-coupling (bold red) and dipolar coupling (bold blue) based polarization transfers. **(B)** Schematic representation of a nucleosome array with H3 tail and core domains colored in red and blue, respectively, in the nucleosome crystal structure. **(C)** ^1^H-^15^N CP-HSQC fingerprint spectra of ^2^H,^13^C,^15^N-H3 in 16-mer nucleosome arrays with 15 bp (red contours), 30 bp (green contours) and 60 bp (blue contours) DNA linkers. The spectrum for arrays with 60 bp DNA linkers shows representative traces along the ^1^H and ^15^N dimensions, and the spectrum for arrays with 15 bp DNA linkers contains an overlay with that for the 60 bp DNA linker sample (single blue contour). All spectra were recorded at 800 MHz ^1^H frequency and 60 kHz MAS rate.

In Figure 1 we show the amino acid sequence of histone H3 and fingerprint 2D ^15^N-^1^H cross-polarization heteronuclear single quantum coherence (CP-HSQC) (Barbet-Massin et al., 2014) solid-state NMR spectra of 16-mer nucleosome arrays with 15, 30 and 60 bp DNA linkers reconstituted with ^2^H,^13^C,^15^N-labeled H3 and back-exchanged in H_2_O, which are nominally expected to contain correlations corresponding to backbone amides of H3 residues ∼45–130 comprising the structured core domain. Most cross-peaks in these spectra were found to have ^1^H chemical shifts in the ∼7–9 ppm regime, consistent with a primarily α-helical structure for the H3 core domain (Figure 1B). In addition, the typical ^1^H and ^15^N linewidths of ∼0.1 and ∼0.4 ppm, respectively, as illustrated in the spectrum for nucleosome arrays with 60 bp DNA linkers suggest a high degree of order at the molecular level for the H3 core domain in the arrays. Cursory visual inspection of the ^15^N-^1^H spectra recorded for arrays with 15, 30 and 60 bp DNA linkers in Figure 1C reveals reasonable overall similarity of ^1^H and ^15^N chemical shifts between all three samples indicative of no large scale structural rearrangements for H3 as a function of DNA linker length. At the same time, the fingerprint spectrum for arrays with 15 bp DNA linkers (Figure 1C; red contours) does show multiple notable ^1^H and/or ^15^N chemical shift differences relative to spectra of arrays with 60 bp (Figure 1C; blue contours) and 30 bp (Figure 1C; green contours) DNA linkers, which point to potential smaller scale local structural differences as discussed in more detail below. In addition, pronounced line broadening, particularly in the ^1^H dimension, is observed for some of the resonances in the spectrum of arrays with 15 bp DNA linkers. The latter is suggestive of increased local heterogeneity at multiple H3 sites relative to arrays with the longer linker DNA lengths, with any associated motions at these sites occuring on the slow-exchange (>ms) time scale.

### Backbone resonance assignments for histone H3 core domain in nucleosome arrays

**FIGURE 2:**
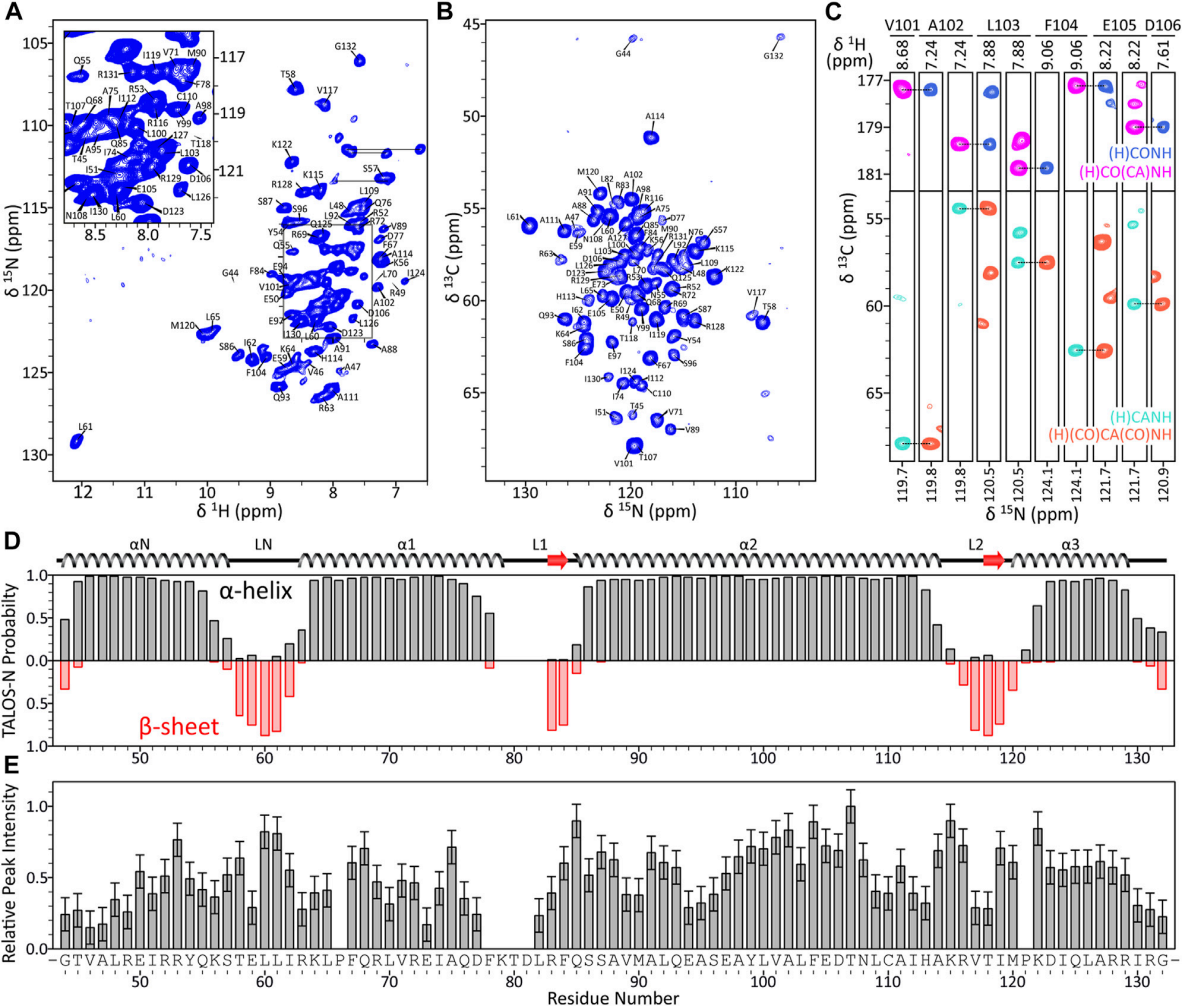
**(A)** Assigned ^15^N-^1^H CP-HSQC spectrum of H3 core domain in 16-mer nucleosome arrays with 60 bp linker DNA length. **(B)** Assigned ^15^N-^13^Cα projection from the 3D (H)CANH spectrum and **(C)** representative small regions from 3D (H)CANH, (H)(CO)CA(CO)NH, (H)CONH and (H)CO(CA)NH spectra of the same sample, illustrating a sequential backbone walk for residues V101-D106. All spectra were recorded at 800 MHz ^1^H frequency and 60 kHz MAS rate. The 2D ^15^N-^1^H spectrum was recorded with acquisition times of 25 ms (t_1_, ^15^N) and 15 ms (t_2_, ^1^H) and a total measurement time of ∼14 h. The 3D ^13^C-^15^N-^1^H spectra were recorded with acquisition times of ∼10 ms (t_1_, ^13^C), ∼12 ms (t_2_, ^15^N), and 30 ms (t_3_, ^1^H) and total measurement times of ∼2–9.5 days. **(D)** TALOS-N secondary structure prediction for the H3 core domain in nucleosome arrays with 60 bp DNA linkers based on the assigned ^1^H, ^15^N and ^13^C chemical shifts with comparison to the secondary structure of H3 in the nucleosome crystal structure (PDB 1KX5), and **(E)** relative peak intensities in the 3D (H)CANH spectrum plotted as a function of residue number.

To establish the ^1^H^N^, ^15^N, ^13^CO, ^13^Cα and ^13^Cβ resonance assignments for histone H3 residues comprising the core domain in nucleosome arrays, we recorded a set of ^1^H-detected 3D solid-state NMR spectra including (H)CANH, (H)(CO)CA(CO)NH, (H)CONH, (H)CO(CA)NH and (H)(CA)CB(CA)NH (Figure 2) for arrays with 60 bp DNA linkers (Barbet-Massin et al., 2014). Overall, signals corresponding to H3 residues in the range 44–132 were detectable in these spectra allowing backbone resonance assignments to be established for 83 out of 87 non-proline residues in this range. The vast majority of these resonance assignments could be unambiguously established from a sequential backbone walk illustrated in Figure 2C with assignments for residues 73 and 82, which displayed attenuated cross-peak intensities, obtained by comparison with characteristic residue-specific chemical shift ranges and with previously reported ^13^C shifts for human H3 in compacted mononucleosomes (Shi et al., 2020a). The experimental chemical shifts were used within the TALOS-N program (Shen and Bax, 2013) to predict the secondary structure for the histone H3 core domain in nucleosome arrays (Figure 2D). This analysis shows four well-defined α-helices with connecting loops, in agreement with the secondary structure of H3 in the nucleosome crystal structure (Davey et al., 2002).

The N-terminal histone H3 tail residues (aa 1–34), most of which could be detected and assigned in J-coupling based solid-state NMR experiments recorded for the 16-mer nucleosome arrays (Zandian et al., 2021), are not observable in CP based spectra of compacted nucleosomes (Xiang et al., 2018; Shi et al., 2020a) and arrays. In addition, signals for residues 35–43 located at the H3 tail-core boundary near the nucleosomal DNA, residues 78–81 located in the L1 loop within the H3 core domain, and C-terminal residues 133–135 were not detectable in either dipolar or J-coupling based solid-state NMR spectra (Figure 2E) likely due to microsecond-millisecond time scale conformational dynamics in the intermediate exchange regime (Shi et al., 2020a; 2020b). We also note the relatively unusual ^1^H and ^15^N chemical shifts for residue L61, both of which are far downfield from the average leucine ^1^H and ^15^N shifts of 8.2 and 121.9 ppm, respectively (Ulrich et al., 2008). These unusual chemical shifts likely result from strong hydrogen bonding interactions involving the L61 amide group and histone H3 residue E97, given the distances of 2.6 Å and 3.1 Å between the L61 nitrogen and E97 side-chain Oε atoms in the nucleosome crystal structure (Davey et al., 2002).

### Comparison of H3 core domain chemical shifts for nucleosome arrays with different DNA linker lengths

Given the overall similarity of ^15^N-^1^H fingerprint spectra for H3 in nucleosome arrays with different linker lengths (Figure 1C), the backbone resonance assignments established for arrays with 60 bp DNA linkers could be readily mapped onto (H)CANH and (H)CONH spectra recorded for arrays with shorter linker lengths. Rudimentary comparison of ^13^Cα chemical shifts for H3 in the different array samples revealed average absolute shift differences for 60 bp vs. 30 bp, 60 bp vs. 15 bp and 30 bp vs. 15 bp of only 0.05 ± 0.05 ppm, 0.08 ± 0.06 ppm and 0.08 ± 0.05 ppm, respectively. Moreover, an analogous comparison involving conserved residues for human H3 in compacted mononucleosomes (Shi et al., 2020a) yielded an average absolute ^13^Cα shift difference of 0.10 ± 0.09 ppm. Since ^1^H^N^, ^15^N and ^13^Cα chemical shifts were available for the vast majority of H3 residues in nucleosome arrays with different DNA linker lengths, to assess in more detail the extent of residue-specific chemical shift variations, for each residue the overall chemical shift difference (Δδ) that accounts for the combined effect of ^1^H^N^, ^15^N and ^13^Cα shift differences was calculated as follows:

$$\Delta \delta ={\left[{\Delta \delta }_{H^{N}}^{2}+{\left(\alpha {\Delta \delta }_{N}\right)}^{2}+{\left(\beta {\Delta \delta }_{C\alpha }\right)}^{2}\right]}^{1/2}$$ (1)

where Δδ_H_^N^, Δδ_N_, and Δδ_Cα_ are the pairwise differences in ^1^H^N^, ^15^N and ^13^Cα chemical shifts between arrays with different DNA linker lengths, respectively, β = 0.3, and α = 0.14 for all residues except glycine and 0.2 for glycines (Williamson, 2013).

**FIGURE 3:**
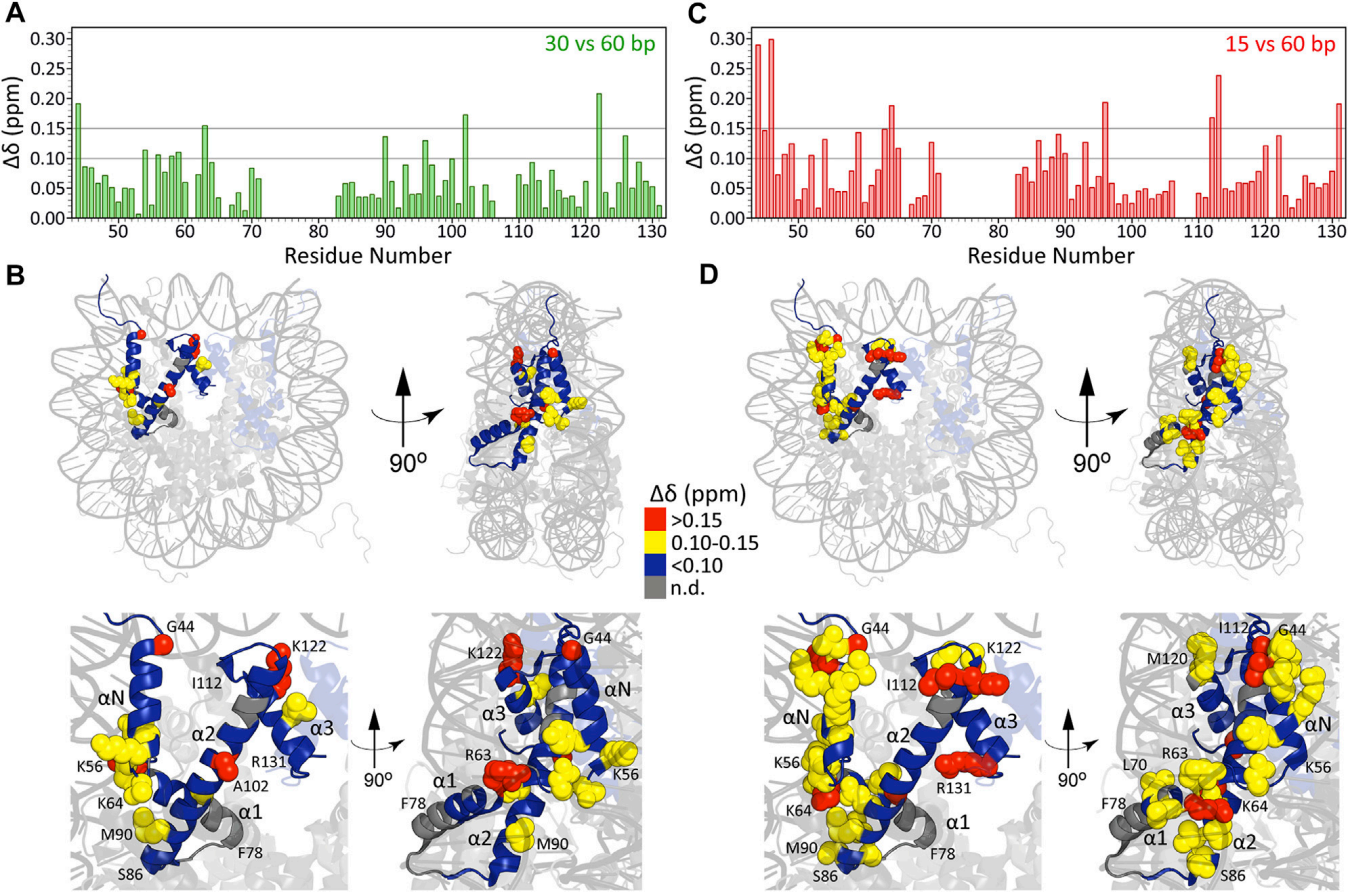
Chemical shift differences (Δδ) for H3 core residues in nucleosome arrays with 30 bp DNA linkers relative to those with 60 bp linkers plotted as a function of residue number **(A)** and mapped on the nucleosome crystal structure **(B)** as described in the inset legend. **(C**, **D)** Same as **(A**, **B)** but for arrays with 15 bp DNA linkers. For both comparisons, the 0.10 and 0.15 ppm thresholds indicated correspond to approximately 2.5 and 5 standard deviations above the average Δδ calculated using subsets of residues with individual Δδ values of less than 0.1 ppm.

In Figures 3A, B we show Δδ values for H3 core residues in arrays with 30 bp and 15 bp DNA linkers relative to arrays with 60 bp DNA linkers as a function of residue number. While on the whole these chemical shift differences are relatively small (average Δδ = 0.07 ± 0.04 ppm and Δδ = 0.09 ± 0.06 ppm, respectively, for arrays with 30 bp and 15 bp DNA linkers vs. 60 bp DNA linkers), on a residue-by-residue basis they were found to be highly non-uniform with the largest Δδ values for both 15 bp vs. 60 bp and 30 bp vs. 60 bp arrays observed for residues located at the H3-nucleosomal DNA interface (Figures 3B, D), and particularly pronounced for arrays with the shortest 15 bp DNA linkers studied (note that the Δδ profile for 15 bp vs. 30 bp arrays was found to quite closely resemble that for 15 bp vs. 60 bp arrays, with average Δδ = 0.10 ± 0.06 ppm). Specifically, for arrays with 30 bp DNA linkers the regions showing the largest chemical shift differences relative to arrays with 60 bp linkers include loop LN between helices αN and α1 and several residues in helices α2 and α3. For arrays with 15 bp DNA linkers, in addition to the above regions, the most significant chemical shift differences relative to arrays with 60 bp linkers expand to include numerous residues in helix αN and clusters of residues in located in helix α1, the base of helix α2 as well as end of helix α2 and loop L2 between helices α2 and α3. Remarkably, many of these residues, including T45, K64 and K122, are involved in key histone-DNA interactions and correspond to sites of PTMs known to impact nucleosome stability (Cosgrove et al., 2004; Tropberger and Schneider, 2010; Bowman and Poirier, 2015). Altogether, these findings are consistent with the notion that relatively short DNA linkers between nucleosomes, which are known to dramatically impact relative nucleosome orientation and chromatin compaction (Correll et al., 2012) and to increase the degree of heterogeneity in nucleosome packing within arrays (Ekundayo et al., 2017), possibly induce DNA unwrapping and considerable strain on the arrays relative to longer DNA linkers. This in turn may lead to side-chains of residues located at the histone core-nucleosomal DNA interface adopting alternate conformations or experiencing shifts in the relative populations of different rotamers. The latter idea is also in line with observed variations in resonance linewidths for different H3 residues, particularly for arrays with 15 bp DNA linkers (c.f. Figure 1C), where residues experiencing the most pronounced line broadening in the ^1^H dimension were generally found to be ones located in the closest proximity to nucleosomal DNA while H3 residues contained within the octamer core appeared as relatively narrow single resonances. Finally, we note that very similar relative integrated cross-peak intensity profiles for the H3 core domain residues (c.f., Figure 2E) were obtained for all nucleosome array samples irrespective of the length of DNA linker regions. These profiles were also generally consistent with that found for human H3 core domain in compacted nucleosomes (Shi et al., 2020b) suggesting that different DNA linker lengths in chromatin do not correlate with large scale changes in μs-ms backbone dynamics for histone H3.

## Concluding remarks

We have established nearly complete assignments of backbone chemical shifts for *Xenopus laevis* histone H3 core residues in large nucleosome arrays with DNA linkers varying between 15 and 60 bp in length. On the whole, the H3 chemical shifts were found to be similar for all nucleosome array samples irrespective of the DNA linker length, and also to shifts reported for human H3 in compacted mononucleosomes, indicative of no major conformational changes in the histone core caused by different length linkers. Notably, however, nucleosome arrays with the shortest 15 bp DNA linkers investigated here showed relatively pronounced differences in chemical shifts and line broadening for numerous residues at the H3-nucleosomal DNA interface consistent with increased heterogeneity in nucleosome packing and structural strain within arrays containing short DNA linkers that likely leads to side-chains of these interfacial residues adopting alternate conformations or experiencing shifts in their rotamer populations compared to arrays with longer DNA linkers. In addition to the findings reported herein, the present study establishes a strong foundation for continued residue-specific investigations of histone protein structure, conformational dynamics and interactions in compacted nucleosomes and nucleosome arrays modeling chromatin at physiological concentrations.

## Materials and methods

### DNA constructs

The DNA constructs used in this study were described previously (Zandian et al., 2021), and consist of 16 tandem repeats of a 147 bp Widom 601 nucleosome positioning sequence (NPS) (Lowary and Widom, 1998) with 15, 30 or 60 bp DNA linkers.

### Histone protein expression and purification

*Xenopus laevis* histones H2A, H2B, H3, and H4 were expressed in *E. coli* BL21(DE3)pLysS cells and purified by gel filtration and ion-exchange chromatography in 7 M urea followed by dialysis against a solution of 2 mM β-mercaptoethanol (BME) as described previously (Luger et al., 1999) and lyophilized. For expression of natural abundance histones H2A, H2B and H4 cells were grown on Luria-Bertani medium, while expression of uniformly ^2^H,^13^C,^15^N-labeled H3 used a D_2_O based minimal medium with ^2^H,^13^C-glucose (3 g/L) and ^15^NH_4_Cl (1 g/L) (Cambridge Isotope Laboratories) as the sole carbon and nitrogen sources, respectively (Gao et al., 2013; Rabdano et al., 2021; Zandian et al., 2021).

### Preparation of histone octamer and nucleosome arrays

Histone octamer containing ^2^H,^13^C,^15^N-H3 was prepared by dissolving the four histone proteins at concentrations of ≤10 mg/ml in 7 M guanidine hydrochloride, 20 mM Tris, 10 mM dithiothreitol, pH 7.5 unfolding buffer in a H2A:H2B:H3:H4 molar ratio of 1.1:1.1:1:1, followed by double dialysis against 10 mM Tris, 1 mM EDTA, 2 M NaCl, 5 mM BME, pH 8.0 refolding buffer. Subsequently, the solution was removed from the dialysis bag, concentrated by using Amicon ultracentrifugal filters (30 kDa cutoff, MilliporeSigma), and purified by gel filtration chromatography in 10 mM Tris, 1 mM EDTA, 2 M NaCl, pH 8.0 buffer as described previously (Luger et al., 1999). Nucleosome array reconstitution was performed as described previously (Zandian et al., 2021) by combining the DdeI digested DNA construct described above and histone octamer in a NPS:octamer molar ratio of 1:1.55 in 5 mM Tris, 0.5 mM EDTA, 2 M NaCl, 1 mM benzamidine hydrochloride hydrate (BZA), pH 8.0 buffer, followed by double dialysis at 4°C against 5 mM Tris, 0.5 mM EDTA, 1 mM BZA, pH 8.0 buffer to remove NaCl. Nucleosome arrays were concentrated using 100 kDa cutoff Amicon ultracentrifugal filters and sucrose gradient centrifugation was performed using sucrose gradients of 5%–40% in 5 mM Tris, 0.5 mM EDTA, pH 8.0 buffer, with fractions containing pure nucleosome arrays combined and exchanged using Amicon ultracentrifugal filters into the final 5 mM Tris, 0.5 mM EDTA, pH 7.0 buffer for solid-state NMR analysis. The formation, purity and saturation of nucleosome arrays with histone octamer were confirmed by using routine electrophoretic mobility shift and atomic force microscopy assays as described in detail previously (Zandian et al., 2021). For the solid-state NMR studies nucleosome arrays in 5 mM Tris, 0.5 mM EDTA, pH 7.0 buffer were precipitated by addition of 5 mM MgCl_2_ according to standard protocols (Hansen, 2002) and transferred by ultracentrifugation to 1.3 mm Bruker zirconia MAS rotors.

### Solid-state NMR spectroscopy

NMR spectra were recorded using a 800 MHz Bruker Avance III HD spectrometer equipped with a 1.3 mm triple-resonance ^1^H-^13^C-^15^N MAS probe. The MAS rate and sample temperature were actively controlled at 60 kHz and ca. 283 K. Sequential resonance assignments were established by using a suite of 3D ^1^H-detected solid-state NMR pulse schemes developed by Pintacuda and co-workers (Barbet-Massin et al., 2014), including (H)CANH, (H)(CO)CA(CO)NH, (H)CONH, (H)CO(CA)NH and (H)(CA)CB(CA)NH. Water suppression was accomplished using a MISSISSIPPI block (Zhou and Rienstra, 2008) with 15 kHz irradiation for 80 m and all experiments used 10 kHz ^15^N and ^13^C WALTZ-16 (Shaka et al., 1983) decoupling. Spectra were processed and analyzed using NMRPipe (Delaglio et al., 1995), nmrglue (Helmus and Jaroniec, 2013) and Sparky (Goddard and Kneller, 2006) software.

## Data Availability

The datasets presented in this study can be found in online repositories. The names of the repository/repositories and accession number(s) can be found below: Chemical shifts for histone H3 core domain in 16-mer nucleosome arrays with 15, 30 and 60 bp DNA linkers have been deposited in the BioMagResBank (https://www.bmrb.wisc.edu) under accession numbers 51,704, 51,705 and 51,706, respectively. Additional contributions presented in the study are included in the article. Further inquiries can be directed to the corresponding author.
